# A Wi-Fi Union Mechanism for Internet Advertising Reciprocal Platform in Microenterprises

**DOI:** 10.3390/s17071617

**Published:** 2017-07-13

**Authors:** Thi Thanh An Nguyen, Che-Pin Chang, Shyan-Ming Yuan

**Affiliations:** 1 EECS International Graduate Programs, National Chiao Tung University, Hsinchu 300, Taiwan; thanhan510@gmail.com; 2 Department of Computer Science, National Chiao Tung University, Hsinchu 300, Taiwan; jalex.cs02g@nctu.edu.tw

**Keywords:** internet advertising, mobile advertising, Wi-Fi advertising, fog computing, QR code

## Abstract

With the evolution of the Internet and smartphone devices, Internet advertising has become one of the most important methods for delivering promotional marketing messages to customers. However, the efficiency of Internet advertising for microenterprise is not very high, since Wi-Fi advertising—which is limited by a small router coverage area—is mainly used. Moreover, because of the lack of money, microenterprises have been using low-cost methods to promote their products. Thus, enhancing the effectiveness of Wi-Fi advertising, and solving the problem of cost and the range of the views are now an essential investigation in this study. In this paper, we propose a reciprocal model with Wi-Fi union mechanism for Internet advertising based on fog computing architecture to enhance the efficiency of advertisement, reduce the cost, and increase the range of the views for microenterprise by using the Internet. In particular, the system was built in advertisers’, publishers’, and consumers’ sides. In our system, we use contribution point (CP) as an exchange value among the participants. Advertisers and publishers can get CP by helping the others in the system to promote their advertisements, increasing their CP by one unit. Similarly, advertisers and publishers can use their CP to ask for assistance from the others, decreasing their CP by one unit. The result shows that the system in a Wi-Fi union is easy to use, and advertisements can be seen by all customers who are using free Wi-Fi from the stores of the union. This method can solve the problem of cost and fixed consumer groups.

## 1. Introduction

Advertising is a form of marketing communication that encourages and persuades for products by acknowledged sponsors by using various media [1]. In global business, advertising can play an important role in addressing the success of a business in which the principles of a successful advertising campaign are high effectiveness, high customer attraction, and low cost. Large enterprises may pay a lot of money promoting their products. In contrast, microenterprises are incapable due to a lack of money [2,3,4].

Microenterprises have used some traditional advertising methods to broadcast advertisements through physical media such as banners, signboards, and flyers [5,6,7]. The cost of these methods including printing and personnel are low and acceptable for microenterprises. Nevertheless, it seems to be ineffective because of the locality and low efficiency, thus requiring the development of a new and innovative strategy for even microenterprises to easily interact with their consumers, to increase the range of the views, to reduce the advertising cost, and eventually to increase their income.

In recent years, Internet advertising which is based on the Internet and mobile devices (e.g., email marketing, web banner advertising, mobile advertising, and so on) has attracted much attention from researchers and developers worldwide, and has changed the approach to advertising [8,9,10,11,12,13,14,15]. Internet advertising has gradually become one of the most potential advertising techniques for not only large enterprises, but also microenterprises. To enhance the benefits of Internet advertising, some techniques have been applied and obtained great achievements, such as search engines [16], social media [17], as well as cloud computing [18]. According to [19], the annual revenues of Internet advertising in the United States have grown from $12.5 billion in 2005 to $59.6 billion in 2015, and the compound annual growth rate (CAGR) over this period for Internet advertising was 17%. Predictably, the spending for Internet advertising will overtake that of TV advertising in the coming years as reported by the eMarketer web site (https://www.emarketer.com/). Among these techniques, social media was one of the hottest developing trends due to its convenience, low-cost, and ease of use. Social networks have varied purposes: connection with friends and family, sharing photos and experiences, meeting new people, advertising products, the creation of new business contacts, and so on [20]. By managing a page on the most popular social networking sites (e.g., Facebook, Twitter, Instagram, etc.), enterprises can post information on new products or activities. The members can hence quickly and easily get the new advertisements. Nonetheless, if the enterprises want to increase the number of the members of their web page, they need to use other advertising methods, such as web banners, flyers, mobile advertising, and so on, which cost a considerable amount of money. Along with social media, cloud computing is also a remarkable developing technology because of less maintenance, continuity, availability, scalability, elasticity, and expert service in shared processing resources and available infrastructure. Recently, cloud computing has led to an emerging interest in developing Internet advertising applications on mobile devices. An application of cloud computing to enable the interactions between m-commerce and billboards through augmented reality was studied [21]. Moreover, in order to keep sight of resource prediction, allocation, advanced reservation, pricing, and refunding, a resource management model of the Cloud of Things to manage resources and advertise services more effectively has been proposed [22]. However, the locality and high latency due to long distance from the infrastructure to cloud computing and the end-users are the disadvantages of using cloud computing techniques; as a result, widespread application in Internet advertising is limited [23]. Fog computing is an extended traditional cloud computing model which can be used to overcome the limitations of cloud computing [24]. In other words, it might be an alternative in reducing data movement across the network, connecting end-users, as well as using mobile devices efficiently. Fog computing was first introduced by the Cisco System to ease wireless data transfer in the Internet of Things (IoT), thus enabling a closer data processing, and solving the challenges of volume, variety, and velocity of the data which are generated by the IoT [25,26]. This technique is a new paradigm that has supported real-time big data analytics, distributed data collection points, and has yielded benefits in advertisements, entertainment, and other applications [27]. Currently, fog computing is widely deployed in various applications and systems, such as smart homes, smart grids, smart vehicles, and health data management [28]. Furthermore, the services of fog for offloading and pre-processing have been used to provide a quick notification of the relevant emergency dealing department [24]. Because of the advantages of fog computing, an Internet advertising application based on fog computing architecture was thus deployed to enhance the effectiveness of advertising for microenterprises in this study.

Recently, Internet advertising applications have been applied in both large enterprises and microenterprises on smart devices. One of the current Internet advertising applications for microenterprises is Wi-Fi advertising, which enables customers to read the advertisement when they are using a free Wi-Fi network from stores, in which the advertising information content will show up as a pop-up window. Because each store has a separate router and the Wi-Fi signal is limited in the coverage area of the router around the store, the advertising effectiveness is not very high. Besides, the use of exchange value among microenterprises to help each other without paying money has not been reported. It is therefore essential to propose a mechanism that allows a group of stores to merge their own Wi-Fi networks into a union system, and to use an exchange value for helping each other in broadcasting advertisements, thus being able to enhance the efficiency of advertisements. Until now, very few studies have investigated Wi-Fi union systems using fog computing and the use of exchange value in the Internet advertising system. The aim of this study is to propose a reciprocal model with a Wi-Fi union mechanism for Internet advertising based on fog computing architecture in order to build an Internet system for microenterprises.

The rest of this paper is organized as follows: Section 2 presents a background of the study, and Section 3 provides our proposed fog computing architecture-based Wi-Fi union mechanism for an Internet advertising system. Section 4 is an evaluation of our system. Finally, conclusions and future works are presented in Section 5.

## 2. Backgrounds

### 2.1. Internet Advertising and Mobile Advertising

Internet advertising (known as online advertising) uses the Internet to deliver advertising messages to customers without a geographical boundary limit. It is one of the most useful methods for enterprises to attract new customers, to increase their revenue, and to extend their reach. There is a cooperation among three sides when using Internet advertising, including the advertisers, the publishers, and the consumers. The advertisers provide the advertisements to be displayed in the online content. The publishers are the web sites or mobile applications in which the advertisements will be integrated into online content. The consumers can read the advertisements from web sites or mobile applications. Some common delivery methods of Internet advertising include email, search engine, social media, display, and mobile advertising.

On the other hand, mobile advertising is a form of Internet advertising which delivers advertisement through wireless mobile devices. It has been rapidly developing along with the explosion of mobile phone and Internet networks [29]. Although mobile advertising has been used for many communication methods and advertising purposes, its method mainly uses two types of communication: push and pull. Push advertising enables the advertising company to send information to the users proactively; pull advertising enables the company to understand more about user behavior through an interactive process by linking users to a web site [30]. Table 1 [30,31] provides comparisons of the characteristics of advertising media. Different from traditional advertising, mobile advertisements and Internet advertisements are interactive and individual which have two-way advertising, high involvement, and easy personalization. Some mobile advertising methods are widely used to deliver promoting advertisements such as SMS/MMS advertising, Mobile Web Banners, and Mobile Web Posters.

### 2.2. Fog Computing

**Figure 1:**
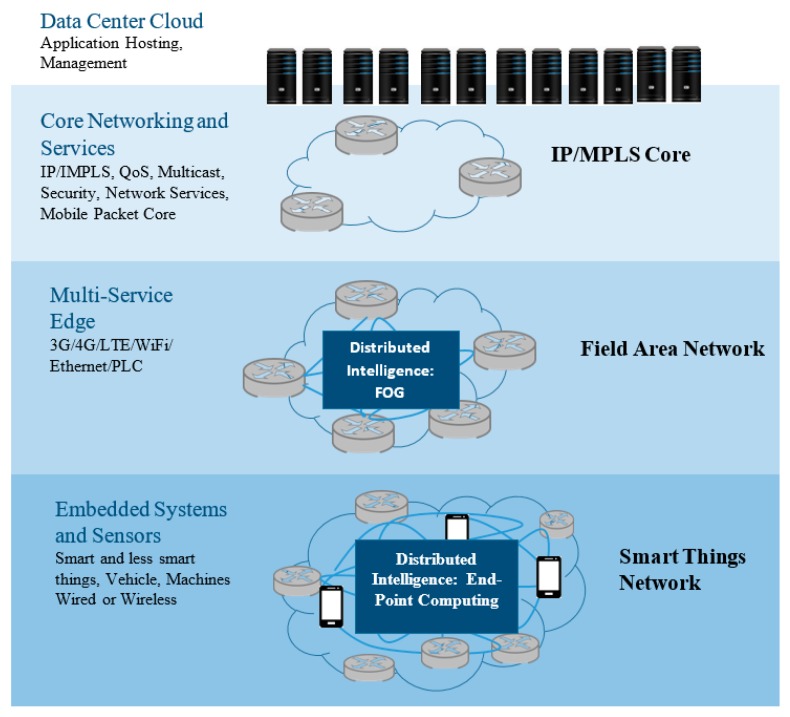
Fog computing architecture (IP/MPLS: Internet protocol/ Multiprotocol Label Switching; LTE: Long-Term Evolution; PLC: power line communication).

Fog computing was introduced by the Cisco system in 2014 dealing with the part of workload and services locally on fog devices (for instance, routers, switches, embedded servers, video surveillance cameras, and industrial controllers), instead of being transmitted to the cloud. As shown in the fog architecture in Figure 1, the new intermediate fog layer is placed between cloud and end-users [32]. The fog layer consists of geo-distributed fog servers, which are highly virtualized computing systems with the large volume data storage, and compute and wireless communication facility. These servers can be deployed anywhere with a network connection, such as stores, restaurants, railways, and vehicles [33]. Due to the fog layer position, the distance between fog server, the end-users, and the cloud layer is only single-hop wireless connection; thus it supports low latency, location awareness, and quality-of-service (QoS) for stream and real-time application [25,34]. Besides, Fog computing is aimed to analyze and process data in devices within the network by running applications rather than in a centralized Cloud; thus, it consequently could deal with the ever-increasing number of connected devices [35].

### 2.3. Related Work

The deployment of Internet advertising has been done by many enterprises such as all the convenience stores of 7-Eleven and FamilyMart located in Taiwan, Coolpon Net in Taiwan and Fon Wireless Ltd. in London, UK.

Several convenience stores in Taiwan have supplied free Wi-Fi advertising platform such as 7-WiFi of the 7-Eleven convenience store system, Fami WiFi of the FamilyMart convenience store system, 1ZFreeWiFi, and so on. Nonetheless, some certain limitations such as the limitation of using time, the frequent appearance of a web banner, and no Wi-Fi access from other services rise inconvenience for consumers. Therefore, it is necessary to design a system that has more flexibility and less restrictions.

Besides, the concept of Wi-Fi sharing has been applied around the globe by Fon Wireless Ltd. company in London, UK, where a Wi-Fi network through “foneras” devices was set up and a member who agreed to share their Wi-Fi signal can connect to other members’ hotspots. Consequently, the benefits of using Wi-Fi sharing might be claimed under four headings, which are (1) to create brand awareness; (2) to enhance consumers’ in-store experience; (3) to increase loyalty; and (4) to improve sales. However, the purpose of Fon Wireless Ltd. is to build a global network by orchestrating small networks, and the users need to pay if they want to use Fon’s services.

Additionally, Coolpon Net is providing a platform for Internet advertising. Microenterprises can create an account as a member in this webpage, and upload their advertisement about image and coupon then. Besides, the microenterprises must spend a certain amount of money to promote their product on this website.

## 3. Fog Computing Architecture-Based Wi-Fi Union Mechanism for Internet Advertising System

**Figure 2:**
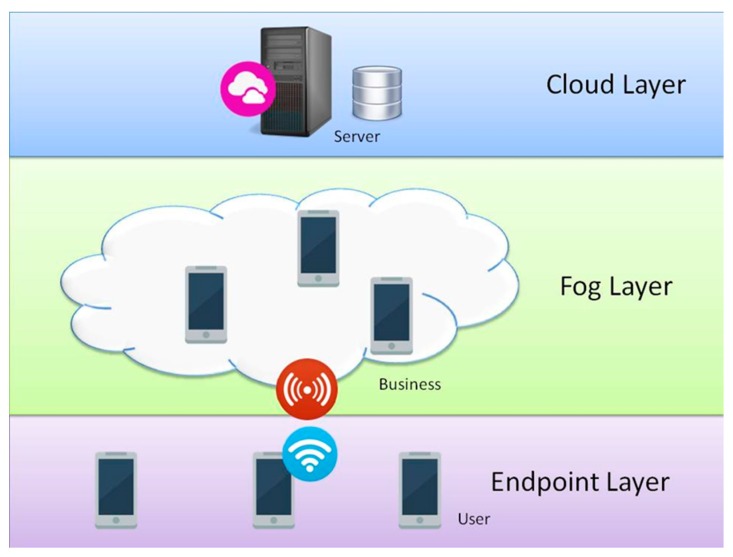
Overview of the Internet advertising system.

The Internet advertising system was built based on Fog computing architecture, as can be seen from Figure 2. The system overview can be classified into three layers: server in cloud layer, advertiser and publisher in fog layer, and consumer in endpoint layer.

### 3.1. Wi-Fi Union Mechanism

The mechanism of a Wi-Fi union is to group different advertisers and publishers, and to merge their own Wi-Fi networks into a union or community. This allows the partners of both advertiser and publisher to share their advertisements to consumers who are using the Wi-Fi network of the advertisers or publishers in the community. Similar to advertiser and publisher, Wi-Fi union is placed at fog layer in the system architecture.

### 3.2. System Architecture

**Figure 3:**
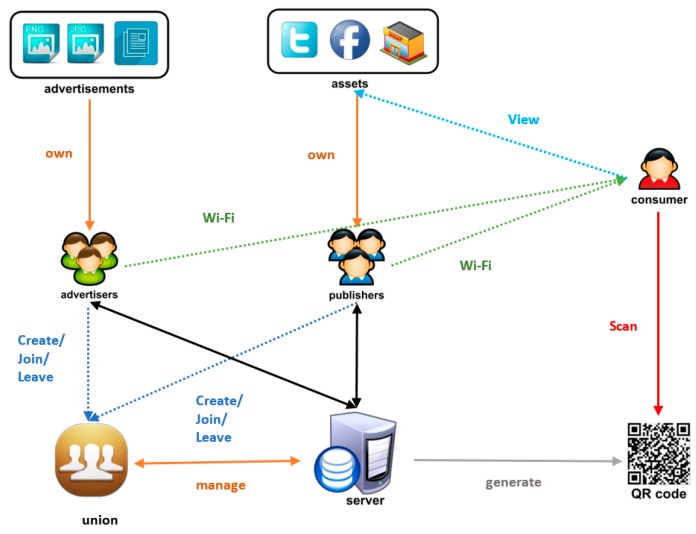
The implementation of an Internet advertising system.

The implementation of the Internet advertising system is described in Figure 3, including the following steps:

1. The Wi-Fi connection and the sending process of an advertisement to a union are directly controlled by an Internet advertising application on the smart devices of the Wi-Fi union members. In addition, an uploading function integrated in this application can be used to send advertisements to the union. Then, any member in the union could broadcast such an advertisement on their website or social network wall as a role of a publisher. When an advertisement is successfully promoted to consumers, the contribution point (CP) values of the publisher increase by one unit, while those of the advertiser decrease by one unit.
2. When a consumer needs to use the free Wi-Fi of any enterprise, the Internet advertising application for the consumer version must be installed into their smart devices. When the consumer logs in, the website of the publisher can appear and the advertisement can be read by the consumer. Full information of the advertisement might be accessed by clicking on the advertisement icon or using scanning a QR Code.

**Figure 4:**
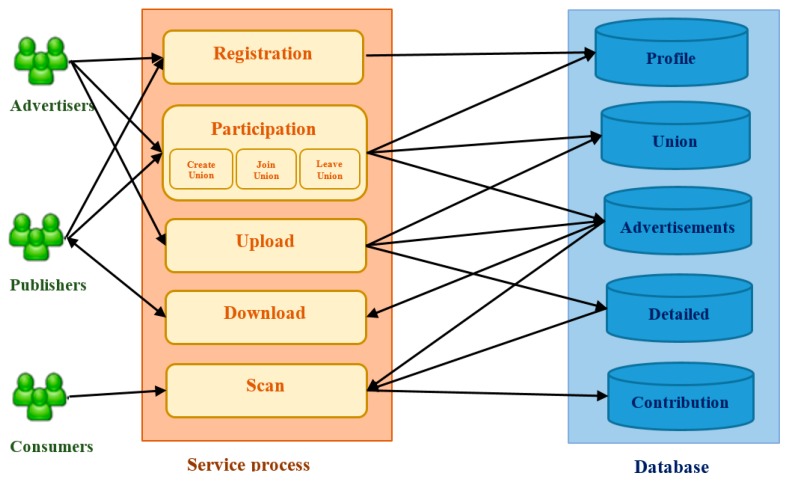
The architecture of Internet advertising system.

Figure 4 shows the architecture of an Internet advertising system. The processes can be listed as follows: registration, participation, upload, download, and scan. The user information such as account, password, and username will be kept in the profile database. The union database is to store the union information including the current list of unions in the system and the list of members in a union. The advertisement and the detailed information of the advertisement are respectively stored in the advertisements and detailed database. Finally, the server has to update the CP of the publisher according to the publisher ID to the contribution database.

#### 3.2.1. Registration Process

**Figure 5:**
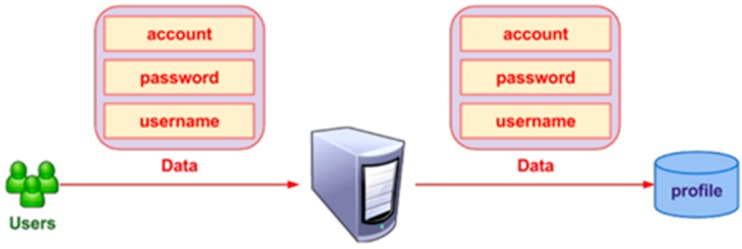
Registration process.

The registration process of Internet advertising system is described in the Figure 5. Firstly, the advertisers and publishers (users) should register on the server by filling in an information form (e.g., account, password, and username). The username is a unique name of each user and is used to identify the other users in the system. The information of the account is henceforth used to log in and stored in the profile database of the server. Any user can view their profile information at any time.

#### 3.2.2. Participation Process

**Figure 6:**
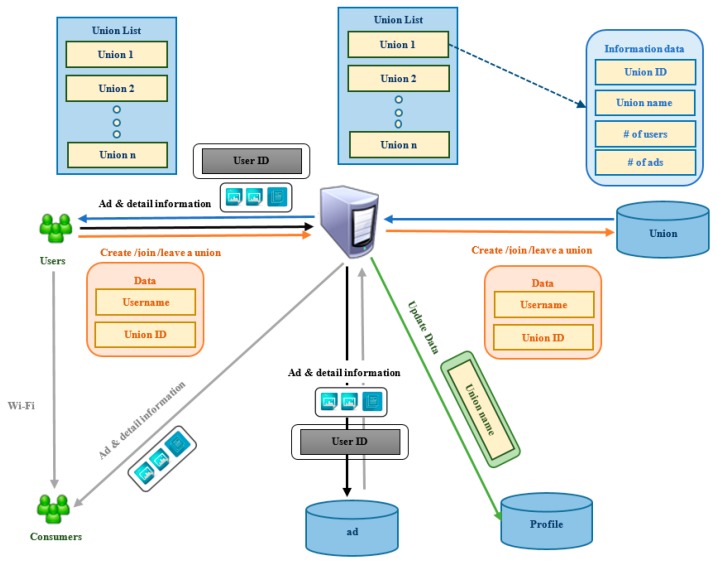
Participation process.

The participation process is shown in Figure 6. First, a user can request a union list from the server with the information union ID, union name, list of users, and list of advertisements in the union. Second, in order to participate in a union, a user will choose which action they want to implement, including: (1) Create a union; (2) Join a union; and (3) Leave a union. Third, the username and union ID will be sent to the server. Herein, username defines who sends the request, and the union ID defines which union the user wants to create, join, or leave. Finally, the server receives the username and union ID by HTTP GET method and updates the community name field of the user in the profile database.

#### 3.2.3. Uploading Process

**Figure 7:**
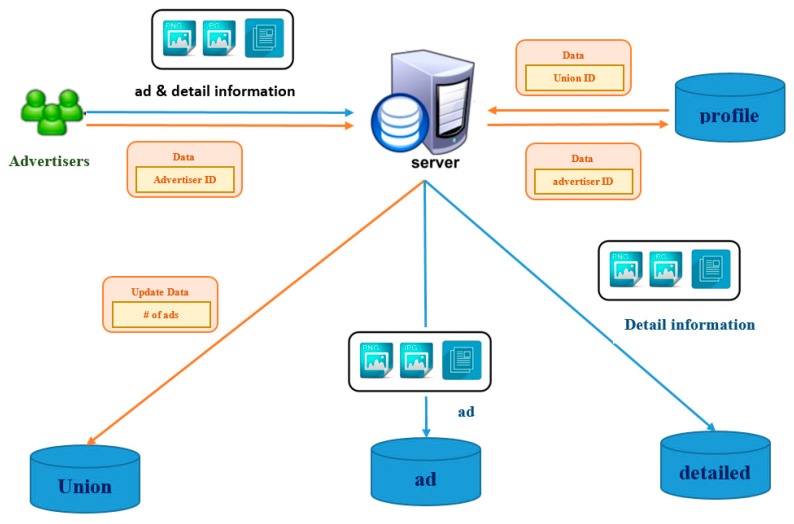
Uploading process.

Figure 7 presents an uploading process of the Internet advertising system. At first, an advertisement and the other detailed information should be prepared to upload to the server. The detailed information includes a coupon, a fan club link, or an official page which can be used to introduce the products or activities to the viewers. Then, the server stores the advertisement and its detailed information in the advertisement database and the detailed database, respectively. Next, the advertiser ID number is used to search the union which the advertiser belongs to. The information of the number of the advertisements could be updated in this union.

#### 3.2.4. Downloading Process

**Figure 8:**
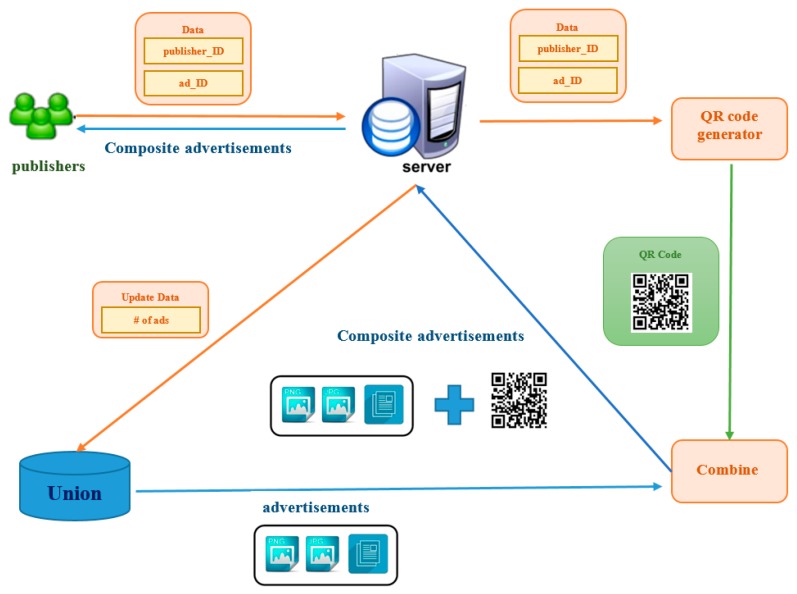
Downloading process.

When a publisher decides to download one advertisement from the union, the publisher will send two parameters to the server through HTTP GET method. The downloading process is shown in Figure 8. Publisher ID describes the person who promotes this advertisement. Ad ID describes the advertisement which the publisher wants to broadcast. In download process, the server will execute two jobs: (1) generating a unique QR code and (2) combining the advertisement with the QR code.

A QR code generator is a website that accepts two parameters: (1) size and (2) content data and returns a QR code. The size of the QR code image can be specified. The minimum value of the size is 10 by 10, and the maximal value is 1000 by 1000. The content is the text or the URL stored within the QR code. Since the consumers scan the QR code to get the advertisement from the server, the URL to the QR code generator can be delivered. Then a QR code can be obtained from the QR code generator. This QR code will be unique because of a unique pair of identification numbers, which consists of publisher ID and advertisement ID.

**Figure 9:**
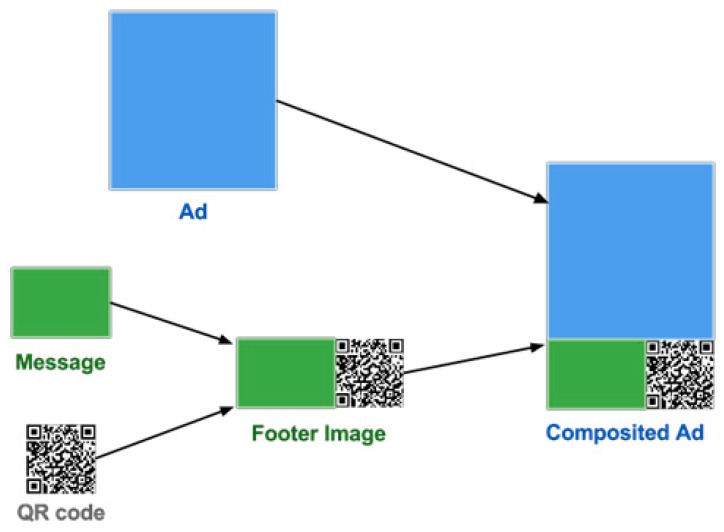
Combining process.

This process needs two pictures: an advertisement image, and a QR code image. The advertisement image can be requested by ad ID from the ad database. The combination process is shown in Figure 9. We modified the pictures by using ImageMagick. We firstly create a footer image which consists of a message and a QR code image. The message describes the use of the QR code. The ad content designed by an advertiser attracts consumers to scan this QR code to obtain more detailed information, such as an image of the menus or a fan club page.

#### 3.2.5. Scanning Process

**Figure 10:**
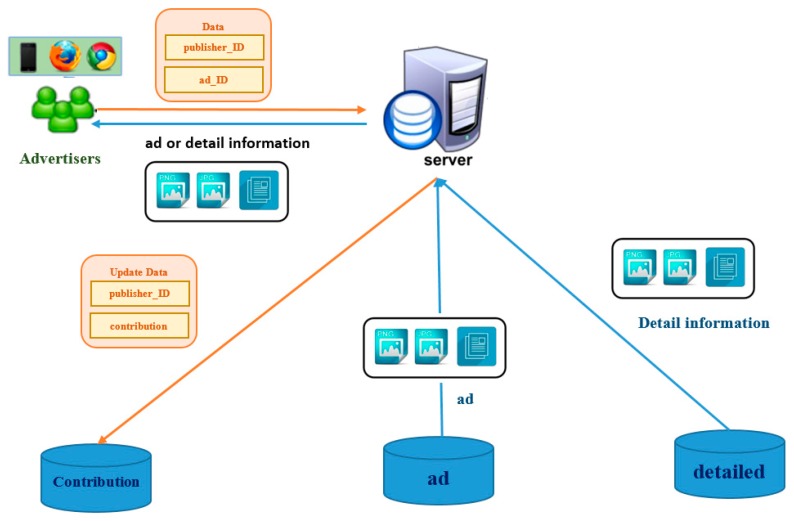
Scanning process.

The scanning process is shown in Figure 10. When a consumer is interested in the advertisement which is hung in the physical shop or posted on the social wall, they may scan the QR code by using the smartphone or browser. They can download the application of the QR code reader on the smartphone or install a plug-in unit into the browser which can scan the QR code.

As a consumer scans the QR code, they will deliver a pair of the ID number of a publisher and an advertisement. The server requests the advertisement and the detailed information from the ad database and the detailed database, respectively. If there is an image of the detailed information, the server returns it to the consumer; otherwise, the server returns the advertisement to the consumer.

Finally, the server has to update the CP of the publisher according to the publisher ID. The calculation of the CP is that if a consumer scans the advertisement once, the CP will add one to itself. The CP describes how many people have scanned this advertisement which the publisher posts.

## 4. Evaluations

The System Usability Scale (SUS) provides a reliable tool and gives a global view of subjective assessments of usability.

To better evaluate the efficiency of the system, SUS is classified into ten questionnaires with five responses from strongly agree to strongly disagree (range from 0 to 4). The odd-numbered questions present the positive meaning, and even-numbered questions present the negative meaning. In order to calculate the SUS score, we first sum the score contributions from each item. Then, the score of odd-numbered question contribution is the scale position minus 1, and the score of the even-numbered question contribution is 5 minus the scale position. Finally, the overall value of SUS is calculated by multiplying the sum of scores by 2.5 [36]. If the SUS score is above 68, it is considered above average. In contrast, an SUS score would be considered below average if its score is under 68 point.

1. I think that I would like to use this system frequently.
2. I found the system unnecessarily complex.
3. I thought the system was easy to use.
4. I think that I would need the support of a technical person to be able to use this system.
5. I found the various functions in this system were well integrated.
6. I thought there was too much inconsistency in this system.
7. I would imagine that most people would learn to use this system very quickly.
8. I found the system very cumbersome to use.
9. I felt very confident using the system.
10. I needed to learn a lot of things before I could get going with this system.

Twenty participants were invited to use and give the feedback in the system, including ten microenterprises (M) (playing the role of advertisers and publishers), and ten customers (C). All are physical participants. The results are shown in Table 2.

The result of ten microenterprises were 72.5, 77.5, 77.5, 70, 72.5, 72.5, 72.5, 70, 75, and 65, and the results of ten consumers were 77.5, 65, 70, 62.5, 80, 65, 70, 75, 70, and 67.5. Based on these results, some conclusions that can be drawn after interviewing are that: (1) almost all advertisers and publishers are satisfied with our system—only one result is lower than average. (2) Some consumers seem to be unsatisfied; they could be disturbed by receiving the advertisements in the notification messages. (3) The question with the lowest score was the fifth one—the advertisers, publishers, and consumers found that some functions in the system were not integrated well. The advertisers and publishers could be confused about uploading and downloading functions because their interfaces are not separated. Moreover, the system has not integrated the QR code function, and the customers need to install the QR code application to scan the QR code in the advertisement in case they are interested, which is inconvenient. We will determine the drawbacks of this system and improve in future works.

## 5. Conclusions and Future Works

A Wi-Fi union mechanism for an Internet advertising reciprocal platform in microenterprises based on fog computing architecture has been proposed in this paper. The main goal of this system is to merger Wi-Fi networks among stores into a union, and to provide a sharing ability between advertisers and publishers by using a credit mechanism called Contribution Point. In addition, an Android application and website platform was built for advertisers and publishers, while an Android application was built for consumers. The results of this study showed that advertisements were sent to all consumers belonging to the union, and the members of the union helped each other to broadcast their advertisements. Thus, the effectiveness of Wi-Fi advertising was enhanced in solving the problem of the cost and the view range. Each microenterprise can promote their product by using Wi-Fi union mechanism or ask for the help from the other microenterprises by using Contribution Point with the lowest cost. It is unfortunate that the participants who joined to evaluate this system are still small, and we may not have evaluated the effectiveness of this system accurately. The role of fog computing has not been clear in our system. Moreover, our system is lacking an authorization mechanism to protect microenterprises and customers from network attacks. In future works, we plan to extend our system to other functions—for example, authorization used to authenticate users, analytics to check the increasing sale figures after using the advertisement and to analyze users’ behavior to improve the efficacy of the advertisement. Besides, in order to show the ability of Fog computing’s role and to increase the reliability of the system, the file transformation function in the Wi-Fi union system can be modified to peer-to-peer transformation or an online hosting service. This will reduce the workload of the server and the response latency.

## Figures and Tables

**Table 1:** Comparisons of advertisement media.

	Magazine/Newspaper	Television	Internet	Mobile
Transmission mode	Visual/haptic	Audio/visual	Audio/visual	Audio/visual/haptic
Presentation type	Pull	Push	Pull	Pull and Push
Involvement	High	Low	Relatively high	High
Communication	One-way	One-way	Two-way	Two-way
Personalization	Medium	Difficult	Easy	Easy

**Table 2:** SUS table. C: customer; M: microenterprise.

Question Item	M1	M5	M10	AVG (M)	C1	C5	C10	AVG (C)
1. I think that I would like to use this system frequently.	4	4	4	3.1	3	4	3	3
2. I found the system unnecessarily complex.	2	1	3	1.6	1	1	2	1.4
3. I thought the system was easy to use.	4	3	4	3.6	4	4	3	3.5
4. I think that I would need the support of a technical person to be able to use this system.	1	2	2	1.3	1	1	1	1.4
5. I found the various functions in this system were well integrated	4	3	4	3.5	3	3	3	2.5
6. I thought there was too much inconsistency in this system	3	2	2	2.8	3	2	3	2.3
7. I would imagine that most people would learn to use the system very quickly.	4	3	4	3.8	4	4	4	3.9
8. I found the system very cumbersome to use.	2	1	1	1.8	1	2	2	1.7
9. I felt very confident using this system.	3	4	3	3.5	3	4	3	3.3
10. I needed to learn a lot of things before I could get going with this system.	1	2	1	1.1	1	1	2	1.3
SUS score	72.5	72.5	65	72.5	77.5	80	67.5	70.25
